# Special Issue *“Redox Homeostasis and Oxidative Stress in Human Metabolism and Disease: 2nd Edition”*

**DOI:** 10.3390/ijms27020724

**Published:** 2026-01-10

**Authors:** Jarosław Nuszkiewicz, Alina Woźniak

**Affiliations:** Department of Medical Biology and Biochemistry, Faculty of Medicine, Ludwik Rydygier Collegium Medicum in Bydgoszcz, Nicolaus Copernicus University in Toruń, 24 Karłowicza St., 85-092 Bydgoszcz, Poland

Restoring and maintaining redox homeostasis is essential for sustaining cellular integrity and metabolic health [1]. The balance between reactive oxygen species (ROS), reactive nitrogen species (RNS), and endogenous antioxidant systems regulates key physiological processes, including mitochondrial bioenergetics, immune responses, metabolic signaling, and tissue remodeling [2]. When this equilibrium is disrupted, oxidative distress arises and contributes to a broad spectrum of human diseases—from metabolic and cardiovascular disorders to neurodegeneration, reproductive dysfunction, chronic inflammation, and tissue injury [3].

Reflecting the central importance of redox biology across these domains, this Special Issue, entitled *Redox Homeostasis and Oxidative Stress in Human Metabolism and Disease: 2nd Edition*, presents an updated and multidisciplinary overview of current advancements in the field. The eleven contributions included here span metabolic health, cardiovascular pathology, pediatric disorders, neurodegeneration, reproductive biology, infectious and inflammatory diseases, toxicology, and tissue protection. Together, they highlight emerging mechanisms, experimental insights, and therapeutic perspectives that continue to shape this rapidly evolving area of molecular science.

## 1. Highlights of This Issue

Wilkinson et al. (Contribution 1) present a comprehensive review of how oxidative and nitrosative distress disrupt skeletal muscle metabolism and insulin signaling in metabolic syndrome and type II diabetes. Their work also highlights how standard pharmacotherapies modulate redox-sensitive pathways, underscoring the need for improved biomarker standardization in metabolic research.

Backston et al. (Contribution 2) summarize the central role of oxidative stress and endothelial dysfunction in the pathogenesis of pediatric hypertension. Their synthesis emphasizes mitochondrial impairment, nitric oxide depletion, and early vascular changes as key mechanisms relevant to risk assessment in young patients.

Baubonis et al. (Contribution 3) report original clinical findings demonstrating that hematological markers linked to inflammatory and oxidative status—particularly the monocyte-to-high-density lipoprotein (HDL) cholesterol ratio—are associated with biochemical and echocardiographic indicators of chronic heart failure severity. These results support the potential use of such markers in personalized cardiovascular evaluation.

Voros et al. (Contribution 4) review the emerging role of ferroptosis—an iron-dependent, lipid peroxidation-driven form of regulated cell death—in ovarian follicular function and dysfunction. Their analysis links ferroptotic pathways to oocyte quality, polycystic ovary syndrome, and outcomes in assisted reproductive technologies.

Castelli et al. (Contribution 5) examine how mitochondrial ROS accumulation, impaired antioxidant capacity, and chronic inflammation contribute to skeletal muscle weakness in neurodegenerative disorders. By integrating findings across disease models, they highlight redox dysregulation as a promising therapeutic target.

Dietrich-Muszalska et al. (Contribution 6) present in vitro data demonstrating the antioxidant effects of curcumin against ziprasidone-induced lipid peroxidation in human plasma. Their results offer insight into the potential mitigation of redox-dependent adverse effects associated with antipsychotic treatments.

Chung et al. (Contribution 7) revisit the gut–liver axis in alcohol-associated disease by emphasizing the underrecognized impact of prolonged intestinal ethanol absorption on systemic oxidative stress. Their review describes how enteric and hepatic redox disturbances synergize to promote inflammation and mitochondrial dysfunction.

Romanowska et al. (Contribution 8) investigate the structural and biochemical consequences of fluoroquinolone exposure on collagen type I in the Achilles tendon. Their synthesis suggests that oxidative stress, altered matrix remodeling, and enzyme dysregulation may underlie fluoroquinolone-associated tendinopathy.

Zenov et al. (Contribution 9) explore how poliovirus infection remodels metabolic pathways in glioblastoma cells to support viral replication and cytopathic activity. Their work highlights the susceptibility of tumor bioenergetics and redox processes to viral manipulation, with implications for future oncolytic strategies.

Bilski et al. (Contribution 10) review evidence linking oxidative stress to inflammatory cascades and tissue destruction in rheumatoid arthritis. They also summarize emerging antioxidant approaches that may complement standard pharmacologic treatments to improve clinical outcomes.

Viebahn-Haensler and León Fernández (Contribution 11) describe ozone-induced redox bioregulation as a potential protective strategy against ischemia–reperfusion injury. Their communication outlines how controlled oxidative preconditioning may preserve mitochondrial integrity and enhance tissue resilience.

## 2. Outlook and Future Directions

Redox biology continues to evolve as a central framework for understanding the molecular underpinnings of human health and disease. Despite significant progress, there remains a pressing need to refine and standardize redox-sensitive biomarkers that can reliably characterize oxidative imbalance across diverse clinical settings. Advances in this area will be essential for improving diagnostic precision and for guiding therapeutic decisions in metabolic, cardiovascular, neurodegenerative, and inflammatory disorders [4].

A deeper exploration of redox-regulated cell death pathways, including ferroptosis and mitochondrial dysfunction, is also likely to yield important mechanistic insights [5]. These processes lie at the intersection of lipid peroxidation, iron handling, and inflammatory signaling, and their clarification will help illuminate how oxidative stress contributes to tissue damage and disease progression. Complementing these mechanistic efforts, emerging therapeutic strategies—from antioxidant adjuvant therapies to redox-modulating interventions and controlled oxidative preconditioning—require rigorous investigation to evaluate their translational potential and long-term safety [6].

Looking forward, interdisciplinary approaches that integrate redox biology with immunology, microbiome research, virology, systems biology, and personalized medicine will be essential for capturing the complexity of oxidative processes in vivo. Strengthening clinically oriented studies, harmonizing methodological approaches, and incorporating standardized outcome measures will further bridge the gap between fundamental discoveries and therapeutic application. Collectively, these efforts are expected to accelerate progress toward innovative strategies capable of restoring redox homeostasis and improving patient outcomes across a wide spectrum of diseases.

## 3. Concluding Remarks

Taken together, the contributions to this Special Issue provide a broad yet coherent overview of how disruptions in redox homeostasis shape human health and disease across multiple biological systems. The articles collectively highlight the mechanistic complexity of oxidative stress, the diversity of its clinical consequences, and the expanding opportunities to translate redox biology into therapeutic innovation. By integrating findings from metabolic, cardiovascular, neurodegenerative, reproductive, infectious, and inflammatory research, this Special Issue underscores the unifying relevance of redox processes in modern molecular medicine. We hope that the insights presented here will stimulate further interdisciplinary investigation and support the development of strategies aimed at restoring redox balance and improving clinical outcomes. It has been our pleasure to curate this collection, and we are confident that it will serve as a valuable resource for researchers working at the interface of redox biology and human disease.

We sincerely thank all the authors for their valuable contributions to this Special Issue and for advancing the scientific understanding of redox biology. We are also grateful to the reviewers for their careful evaluations and constructive feedback, which greatly improved the quality of the published articles. Finally, we extend our appreciation to the Editorial Office of the *International Journal of Molecular Sciences* for their continuous support throughout the preparation of this Special Issue.

## Data Availability

No new data were created or analyzed in this study. Data sharing is not applicable to this article.
